# Continuous up to 4 Years Entecavir Treatment of HBV-Infected Adolescents – A Longitudinal Study in Real Life

**DOI:** 10.1371/journal.pone.0163691

**Published:** 2016-09-29

**Authors:** Małgorzata Pawłowska, Krzysztof Domagalski, Beata Smok, Paweł Rajewski, Magdalena Wietlicka-Piszcz, Waldemar Halota, Andrzej Tretyn

**Affiliations:** 1 Department of Paediatric Infectious Diseases and Hepatology, Faculty of Medicine, Collegium Medicum, Nicolaus Copernicus University, Bydgoszcz, Poland; 2 Centre for Modern Interdisciplinary Technologies, Nicolaus Copernicus University, Toruń, Poland; 3 Provincial Infectious Diseases Hospital, Bydgoszcz, Poland; 4 Department of Theoretical Foundations of Biomedical Sciences and Medical Information Technology, Faculty of Pharmacy, Collegium Medicum, Nicolaus Copernicus University, Bydgoszcz, Poland; 5 Department of Infectious Diseases and Hepatology, Faculty of Medicine, Collegium Medicum, Nicolaus Copernicus University, Bydgoszcz, Poland; 6 Department of Plant Physiology and Biotechnology, Nicolaus Copernicus University, Toruń, Poland; Instituto de Diagnostico y Referencia Epidemiologicos, MEXICO

## Abstract

This study evaluated the long-term (up to 4 years) efficacy and safety of entecavir ETV treatment and analysed the significance of baseline and on-treatment factors in long-term ETV outcomes in adolescents with chronic hepatitis B (CHB). We determined the cumulative virological and serological outcomes of 44 adolescents with CHB receiving ETV for up to 4 years. To investigate the dynamics of HBV DNA, ALT activity and hepatitis B e antigen (HBeAg) seroconversion over time and their associations with the considered factors, generalized estimating equation (GEE) models were used. The cumulative rates of undetectable HBV DNA (<20 IU/ml) and HBeAg seroconversion after 4 years were 89.7% and 55.4%, respectively. In the study group, we showed that having undetectable HBV DNA at the 6^th^ or 12^th^ month of therapy predicted the achievement of a sustained response rate (SRR, defined as the loss of HBV DNA, loss of HBeAg and ALT normalization) at year 3 of ETV therapy (*P* = 0.048, OR = 5.83; *P* = 0.012; OR = 14.57, respectively). The GEE analysis indicated that of the different factors, the duration of ETV therapy had a strong impact on the achievement of virological suppression, HBeAg seroconversion and SRR in adolescents. Each month after the initiation of therapy, the odds of loss of HBV DNA increased by approximately 5% (OR = 1.05, *P*<0.0001), on average. Additionally, the GEE analysis revealed that adolescents with an age at infection of ≥10 years had 3 times higher odds of achieving undetectable HBV DNA than patients with a younger infection age (OR = 3.67, *P* = 0.028). None of the ETV-treated patients reported significant adverse effects. ETV is an effective and safe treatment option for adolescents with CHB. Undetectable HBV DNA in the 6^th^ and/or 12^th^ month of ETV treatment and older age at infection could predict maintained virological suppression.

## Introduction

Hepatitis B virus (HBV) infection is still one of the most important infectious diseases, with 2 billion people infected worldwide and more than 600,000 deaths each year [1]. Despite the availability of effective and safe vaccination programs, chronic HBV infection constitutes a serious public health problem. Children infected with HBV perinatally or in the first year of life have a high risk of chronicity, with the development of severe clinical consequences such as liver cirrhosis or hepatocellular carcinoma (HCC).

Among children, HBV infection concerns those who have not been vaccinated at all or have not gone through the full cycle of vaccinations, those who were exposed to HBV prior to vaccination or were born to HBV-infected mothers and did not receive immuno-prophylaxis. However, Haber et al. observed the occurrence of vertical HBV infection in 5% of infants despite appropriate immuno-prophylaxis and vaccination [2]. HBV infections in young children often involve a long phase of immunotolerance in which patients have a high viral load, HBeAg presence and normal ALT activity.

The goal of hepatitis B treatment is to reduce viral replication and to minimize hepatic injury. To date, five medications for the treatment of children/adolescents with chronic hepatitis B (CHB) have been approved. These are interferon-alpha, lamivudine, adefovir, entecavir and tenofovir [3].

The treatment of CHB in children and adolescents is controversial due to its unsatisfactory results in immune-tolerant states and the risk of resistance [4, 5]. Some children with CHB require antiviral treatment to prevent liver cirrhosis and HCC. The qualification for therapy is based on the results of alanine aminotransferase (ALT) activity, HBV DNA levels, hepatitis B e antigen (HBeAg) presence and liver histology. Of particular importance seems to be the presence of co-existing liver disease and a family history of cirrhosis and/or HCC [6].

The aim of this study was to evaluate the long-term efficiency and safety of entecavir (ETV) treatment in children and adolescents infected with HBV. This is a unique analysis of the long-term 4-year treatment of adolescents with ETV in a real-life setting. The results of this study may be useful in enhancing the importance of ETV treatment in clinical practice as one of the available therapeutic options for adolescents with CHB.

## Patients and Methods

### Study population and clinical evaluation

In our study, 57 patients were evaluated. We excluded 13 patients who were treated for less than 12 months or who didn’t have the necessary medical documentation. Patients with histological evidence of HCC or a chronic liver disease other than CHB and those who were co-infected with HCV or HIV were excluded. There were no pregnant women in the study group. Our study did not include patients in the immunotolerant phase. Overall, we collected data from 44 consecutive patients who, in most cases, were infected with HBV in childhood and had begun treatment with ETV at the Department of Paediatric Infectious Diseases and Hepatology. All examined patients were treated with ETV monotherapy at dosages of 0.5 mg/day or 1 mg/day for at least 48 weeks. The eligibility criteria were having detectable serum HBsAg for 6 months and the presence of two of the following criteria: detectable HBV DNA in the serum, increased ALT activity and histopathological changes in the liver. In all patients, a liver biopsy and liver ultrasound were performed at baseline. The liver biopsy specimens were assessed according to the modified Scheuer scale (grading from 0–4 and staging from 0–4) [7]. This study included 30 children who were previously administered antiviral treatment; 9 with interferon (IFN), 5 with lamivudine (LAM) and 16 with IFN and LAM. In all patients treated with LAM, we assessed the presence and the profile of HBV resistance mutations against LAM. We detected M204V, J80, M204J, L180M, S202G, L80V, M204, L180, M204I and M180M mutations. Only patients with no presence of mutations against LAM before the start of ETV treatment were included in the study.

In the analysed group of patients, the age at HBV infection ranged from 4 months to 14 years (median 3 years). In 38 patients, HBV infection was recognized before the 10^th^ year of age, and in 6, at ≥10 years of age. Information about the occurrence of infection at ≥10 years of age was based on medical records, which negated the presence of HBsAg and anti-HBc in these patients before they were 10 years old. The median age at the start of ETV treatment was 16 years. We did not use any specific age to begin treatment. Although ETV therapy was only recommended in children older than 16 years (FDA and ESPGHAN recommendation), this study included patients younger than 16 years for ETV therapy. Every six months of treatment, the patients were required to return to the clinic to receive a physical examination and laboratory tests for serum HBV DNA levels, presence of serum HBeAg and HBsAg and ALT activity and to report adverse events.

The effectiveness of the treatment was evaluated on the basis of the patient’s virological, serological and biochemical response, which was assessed by analysing HBV DNA levels, HBeAg status and ALT activity before and during the treatment (at weeks 4, 12 and 24 and every six months until month 48). Furthermore, the combined response was assessed. The effectiveness of the treatment was evaluated based on the sustained response rate (SRR), defined as the suppression of viral replication to undetectable HBV DNA levels (<20 IU/mL), HBeAg seroconversion to anti-HBe (HBeAb), and normalization of ALT activity (<40 IU/L) in HBeAg-positive (HBeAg(+)) patients [8]. In the HBeAg-negative (HBeAg(-)) patients, SRR was defined as an undetectable HBV DNA level and ALT normalization. HBV DNA level was assessed by quantitative polymerase chain reaction assay (COBAS AmpliPrep/COBAS TaqMan HBV Test; Roche Diagnostics). Virological breakthrough was defined as either a >10-fold increase in serum HBV DNA from the nadir for patients with detectable HBV DNA or HBV DNA >200 IU/ml after HBV DNA clearance (>10-fold increase from the limit of detection, 20 IU/ml).

Observations up to month 48 were not available for all patients. For patients who started ETV therapy later, the period of therapy was shorter, and data up to the 48^th^ month were unavailable. Thus, the number of observations at the considered time points decreased from 44 at baseline to 12 months to 41 at the 24^th^ month, 31 at the 36^th^ month, and 23 at the 48^th^ month.

### Ethical aspects

The protocol was approved by the NCU Bioethics Committee at the Collegium Medicum NCU. All procedures conformed to the ethical guidelines of the 1975 Declaration of Helsinki. All patients older than 16 years and all legal guardians signed a written informed consent.

### Statistical analysis

The summary statistics are presented as medians with interquartile ranges or absolute and relative frequencies, n (%), as appropriate. Differences between continuous variables were analysed by Mann-Whitney U test, and differences in categorical variables were tested using chi-square or Fisher’s exact test. The concentration of HBV DNA was analysed as a binary variable (undetectable and ≥20 IU/mL). The ALT activity was also considered a categorical variable (normal ALT activity (<40 IU/L) vs elevated ALT activity (≥40 IU/L)). The long-term treatment effects during the 4-year period of ETV therapy were analysed using generalized estimating equations (GEE), which account for the correlation between repeated observations from the same individual at multiple time points. To model the progression in virological suppression to HBV DNA levels <20 IU/mL, normalization of ALT activity, HBeAg seroconversion and SRR during ETV therapy, a GEE logistic regression model with logit link and an autoregressive working correlation structure was fitted to the data. Initially, the time of ETV therapy, HBeAg status at baseline, HBV DNA viral load, ALT activity at baseline, gender, previous NA-therapy, grading, staging, age at infection and age at treatment start were included in the model as covariates. In the analysis, all patients’ HBV DNA levels at baseline were determined and described by the percentage of patients in established categories: </≥2,000 or </≥ 20,000 IU/ml. On the basis of the Wald statistic, forward elimination was performed, and finally, the model with significant covariates was fitted to the data. The cumulative rates of virological suppression, HBeAg seroconversion and SRR achievement were estimated using the Kaplan-Meier method, and differences between treatment subgroups were calculated by the log-rank test. The results were considered statistically significant when the *P*-value was less than 0.05. The statistical analysis was performed with the use of R-software, version 3.0.3. (package gee) [9].

## Results

### Baseline characteristic

This study recruited HBeAg(+) (47.7%) and HBeAg(-) (52.3%) CHB patients. The characteristics of the examined patients in the whole group and stratified by HBeAg status at baseline are presented in Table 1. The examined group of patients was predominantly male (37/44–84%) and patients infected with HBV before the age of 10 years (38/44–86%). All patients who were ≥10 years at infection were HBeAg-negatives. The pretreatment liver biopsy sample data, showed that most patients had stage F1 (38.6%) and F2 (38.6%) fibrosis and grade A2 (47.7%) inflammation, according to the modified Scheuer score. In 24/44 (54.6%) patients, HBV DNA ≥20,000 IU/mL was observed. Most HBeAg(-) patients (66.7%) had baseline HBV DNA levels under 20,000 IU/mL, and most HBeAg(+) patients had baseline HBV DNA levels above 20,000 IU/mL (73.9%).

**Table 1:** Patient characteristics in the whole group and according to HBeAg status.

Characteristic		All patients	HBeAg(-)	HBeAg(+)
N		44	21	23
Age [yr]		16 (14.5–17)	17 (15–17)	15 (11–16)
Gender	Male	38 (86.4)	16 (76.2)	22 (95.7)
	Female	6 (13.6)	5 (23.8)	1 (4.3)
Previous NA-therapy	No	23 (52.3)	13 (61.9)	10 (43.5)
	Yes	21 947.7)	8 (38.1)	13 (56.5)
Age at infection, [yr]	<10	38 (86.4)	15 (71.4)	23 (100.0)
	≥10	6 (13.6)	6 (28.6)	0 (0.0)
ALT [IU/L]		33.5 (22.0–97.0)	27.0 (20.0–58.0)	64.0 (28.0–105.0)
ALT [IU/L]	<40	15 (34.1)	9 (42.9)	6 (26.1)
	≥40	29 (65.9)	12 (57.1)	17 (73.9)
HBV DNA viral load [IU/ml], log10		4.15 (2.9–7.3)	3.07 (2.4–4.3)	7.23 (3.8–8.0)
HBV DNA viral load [IU/ml]	<2,000	16 (36.4)	12 (57.1)	4 (17.4)
	2,000–20,000	7 (15.9)	4 (19.0)	3 (13.0)
	≥20,000	21 (47.7)	5 (23.8)	16 (69.6)
Grading	0	3 (6.8)	3 (14.3)	0 (0.0)
	1	13 (29.6)	7 (33.3)	6 (26.1)
	2	21 (47.7)	8 (38.1)	13 (56.5)
	3	4 (9.1)	2 (9.5)	2 (8.7)
	4	3 (6.8)	1 (4.8)	2 (8.7)
Staging	0	3 (6.8)	2 (9.5)	1 (4.4)
	1	17 (38.6)	5 (23.8)	12 (52.2)
	2	17 (38.6)	11 (52.4)	6 (26.1)
	3	6 (13.7)	2 (9.5)	4 (17.4)
	4	1 (2.3)	1 (4.8)	0 (0.0)

All data are presented as the median (interquartile) or number (%); ALT, alanine aminotransferase; NA, nucleotide analogue; staging and grading were assessed according to a modified Scheuer scoring system.

### Virological, biochemical and serological responses

**Fig 1:**
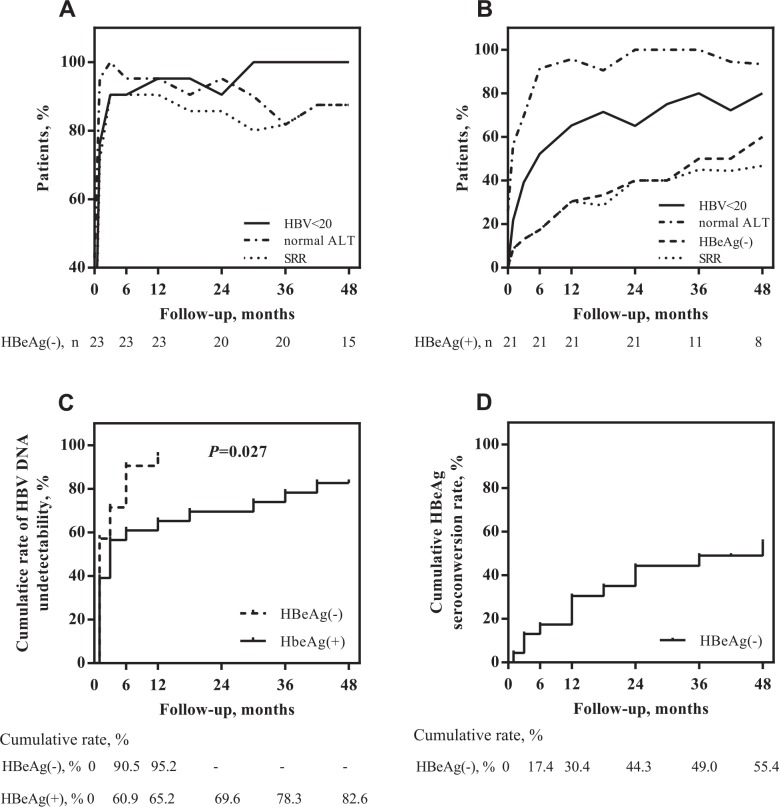
Longitudinal changes in virological, biochemical and serological responses in subsequent months of ETV therapy in HBeAg(+) and HBeAg(-) patients at baseline. Frequencies of HBV DNA <20 IU/mL, normal ALT activity (<40 IU/L), loss of HBeAg and achievement of SRR during ETV therapy in HBeAg(-) (A) and HBeAg(+) (B) patients were calculated. Cumulative rates of viral suppression to undetectable HBV DNA (<20 IU/mL) were determined in patients stratified by HBeAg status (C). The cumulative rate of HBeAg seroconversion was calculated in the subgroup of HBeAg(+) patients (D).

The percentages of baseline HBeAg(-) (Fig 1A) and HBeAg(+) (Fig 1B) patients with undetectable serum HBV DNA (<20 IU/mL), loss of HBeAg and normal ALT activity in subsequent months of therapy are shown. Regardless of HBeAg status, the number of patients with HBV DNA <20 IU/mL gradually increased in the subsequent months of ETV therapy. The highest increase in the number of patients with HBV <20 IU/mL was observed during the first six months of therapy. In this period of time, the number of patients with HBV DNA <20 IU/mL increased to 75.0% (to 52.0% and to 90.5% in the HBeAg(+) and HBeAg(-) patients, respectively). At 12 months of therapy, 95.2% of HBeAg(-) and 65.2% of HBeAg(+) patients had HBV DNA <20 IU/mL (Table 2). In the HBeAg(+) group, the percentage of patients with HBV DNA <20 IU/mL increased gradually to 12/15 (80.0%) at month 48. In the group of HBeAg(-) patients treated with ETV for at least 30 months, 100% of the patients were observed to have HBV DNA <20 IU/mL.

**Table 2:** Treatment responses at 12 months of therapy in the whole group and according to HBeAg status.

Characteristic		All patients	HBeAg(-)	HBeAg(+)
N		44	21	23
HBV at 12 months, [IU/ml]	<20	35 (79.5)	20 (95.2)	15 (65.2)
	≥20	9 (20.5)	1 (4.8)	8 (34.8)
HBeAg at 12 months	HBeAg(-)	28 (63.6)	21 (100.0)	7 (30.4)
	HBeAg(+)	16 (36.4)	0 (0.0)	16 (69.6)
ALT at 12 months [IU/L]	<40	42 (95.45)	20 (95.2)	22 (95.7)
	≥40	2 (4.5)	1 (4.8)	1 (4.3)
SRR at 12 months	SRR	26 (59.1)	19 (90.5)	7 (30.4)
	No SRR	18 (40.9)	2 (9.55)	16 (69.6)

SRR was defined as HBV DNA <20 IU/mL, ALT <40 IU/L, and HBeAg(-).

For all patients, the cumulative rates of virological suppression to HBV DNA <20 IU/mL were 79.5%, 82.1%, 87.2% and 89.7% for years 1, 2, 3 and 4, respectively. The cumulative rates of HBV <20 IU/mL in patients stratified according to HBeAg status were shown in Fig 1C. HBeAg(-) patients achieved a significantly higher cumulative rate of HBV DNA <20 IU/mL compared to HBeAg(+) patients (*P* = 0.027). The final cumulative rates of HBV <20 IU/mL were 95.2% for HBeAg(-) patients at year 1 and 82.6% for HBeAg(+) patients at year 4.

In 29/44 (65.9%) patients, increased ALT activity was observed (73.9% in HBeAg(+) patients at baseline and in 57.1% patients who were HBeAg(-) at baseline). The highest increase in ALT <40 IU/L was observed in the first 6 months of therapy, and in the 12^th^ month, elevated ALT activity was only present in 2/44 patients (4.6%) (Table 2). Then, the trend seemed to stabilize, and fluctuations in elevated ALT activity ranging between 2.5 and 9.5% were observed in the whole group of patients.

In the HBeAg(+) group (23 patients), HBeAg/anti-HBe seroconversion occurred in 12 patients (52.2%): in the first year of ETV therapy for 7 patients, in the second year of therapy for 3 patients and in the third and fourth year, single cases of seroconversion occurred. The cumulative rate of HBeAg seroconversion up to year 4 was 55.4% (Fig 1D). None of the analysed patients achieved HBsAg seroclearance.

**Fig 2:**
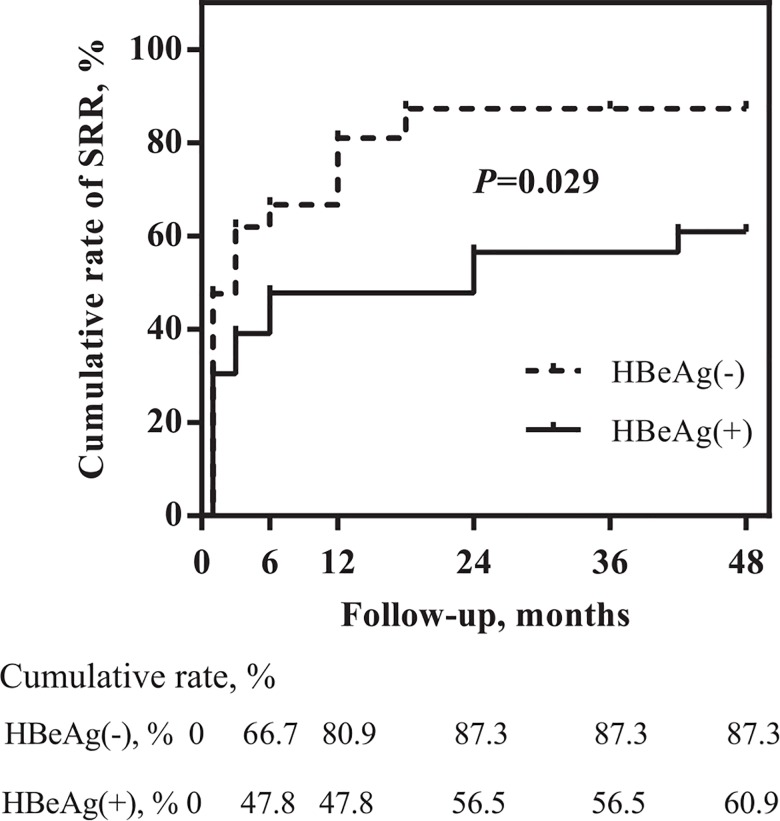
Cumulative rate of SRR achievement in patients with ETV therapy by HBeAg status at baseline. SRR (sustained response rate) was defined as HBV DNA <20 IU/mL, loss of HBeAg and normalization of ALT activity (<40 IU/L) in patients HBeAg(+) or as undetectable HBV DNA <20 IU/mL with normalization of ALT activity in HBeAg(-) patients.

Finally, the dynamics of the changes over time in SRR achievement were studied. The percentages of patients with SRR during ETV treatment are shown in Fig 1A and 1B. The highest increase in the number of patients with SRR was observed during the first six and twelve months of therapy in HBeAg(-) and HBeAg(+) patients, respectively. In contrast to HBeAg(+) patients, in whom we observed a successive slight increase in SRR in the subsequent months of ETV therapy, the trend seemed to stabilize in HBeAg(-) patients; however, some fluctuations were observed, which resulted mainly from changes in ALT activity. The cumulative rate of SRR achievement is shown in Fig 2. HBeAg(-) patients achieved a significantly higher cumulative rate of SRR when compared to HBeAg(+) patients (*P* = 0.029).

### Factors associated with the loss of HBV DNA during ETV treatment

GEE logistic regression was used to model the evolution in loss of HBV DNA expressed as a binary variable (<20 IU/mL *vs* ≥20 IU/mL) during ETV therapy. Based on the Wald statistic, the final model with the covariates time of ETV therapy, HBeAg status at baseline, HBV DNA expressed as a binary variable (cut-off of 20,000 IU/ml) and age at infection was fitted to the data (Table 3). GEE analyses have shown that the variable determining the presence of previous NA-therapy has no significant effect on virological suppression. The obtained estimates indicate that the probability of the loss of HBV DNA is associated with the length of the treatment period and that the passage of each month during therapy increases the odds of the loss of HBV DNA by approximately 5% (OR [odds ratio] = 1.05, [95% confidence internal] 95% CI = 1.03–1.07). The presence of HBeAg(+) at baseline decreased the odds of the loss of HBV DNA (OR = 0.19 (0.08–0.47)), and the presence of a HBV DNA viral load ≥20,000 IU/mL decreased the probability of the loss of HBV DNA as well (OR = 0.22 (0.08–0.59)). A higher infection age (≥10 years of age) increased the probability of the loss of HBV DNA (OR = 3.67 (1.15–11.76)) at any time point. Analyses of the HBeAg(+) and HBeAg(-) subgroups showed that time of ETV therapy was the most important factor in the elimination of HBV DNA and showed that a HBV DNA viral load ≥20,000 IU/mL was a more important covariate in the HBeAg(+) than in the HBeAg(-) subgroup. Additionally, the analysis of the HBeAg(-) subgroup confirmed the importance of infection age on the elimination of HBV DNA.

**Table 3:** Factors associated with the loss of HBV DNA and normalization of ALT activity (multivariate GEE models).

Covariate	Loss of HBV DNA		Normalization of ALT activity	
	OR (CI)	*P* value	OR (CI)	*P* value
All patients				
Time, per month	1.05 (1.03–1.07)	<0.001	1.039 (0.99–1.08)	0.067
Baseline HBeAg, positive	0.19 (0.08–0.47)	<0.001	-	-
Age at infection, ≥10 years	3.67 (1.15–11.76)	0.028	-	-
HBV DNA, ≥ 20,000 IU/mL	0.22 (0.08–0.58)	0.003	-	-
HBeAg(+) group				
Time, per month	1.06 (1.03–1.09)	<0.001	1.111 (0.99–1.24)	0.053
HBV DNA, ≥ 20,000 IU/mL	0.14 (0.03–0.64)	0.011		
HBeAg(-) group				
Time, per month	1.15 (1.04–1.27)	0.008	-	-
Age at infection, ≥10 years	2.34 (1.04–5.28)	0.040	-	-
HBV DNA, ≥ 20,000 IU/mL	0.50 (0.24–1.05)	0.069	-	-

Loss of HBV DNA, HBV DNA <20 IU/ml; normalization of ALT activity, ALT < 40 IU/ml; OR (CI), odds ratio with 95% confidence interval; Em dash (-), covariate not included in final model.

### Factors associated with the normalization of ALT activity during ETV therapy

To study the association between the incidence of normal ALT activity and the considered covariates, the GEE model was fitted to the data. Table 3 contains the estimates of the final model. We found no significant factors associated with ALT normalization in the whole group and HBeAg(+) and HBeAg(-) subgroups. Regardless of the study group, the duration of ETV treatment had no significant effect on the normalization of ALT activity; however, the marginal p-value may suggest a potential association of the duration of ETV treatment with normalization of ALT activity.

### Factors associated with the achievement of HBeAg seroconversion and SRR

The time of therapy and HBV DNA ≥20,000 IU/mL were included as significant covariates in the model assessing the achievement of HBeAg seroconversion in the HBeAg(+) subgroup (Table 4).

**Table 4:** Factors associated with the loss of HBeAg(-) and attainment of SRR (multivariate GEE models).

Covariate	Loss of HBeAg		SRR	
	OR (CI)	*P* value	OR (CI)	*P* value
All patients				
Time, per month	NA	NA	1.03 (1.01–1.06)	0.007
Baseline HBeAg, positive	NA	NA	0.06 (0.02–0.18)	<0.001
HBeAg(+) group				
Time, per month	1.03 (1.012–1.05)	0.001	1.04 (1.02–1.06)	0.007
HBV DNA, ≥ 20,000 IU/mL	0.19 (0.05–0.67)	0.009	-	-
HBeAg(-) group				
-	NA	NA	-	-

SRR, HBV DNA <20 IU/mL, ALT <40 IU/L and HBeAg(-); OR (CI), odds ratio with 95% confidence interval; Em dash (-), covariate not included in final model.; NA, not applicable

The obtained estimates indicate that each month of therapy increased the odds of the loss of HBeAg by approximately 3% (OR = 1.03 (1.01–1.05)). According to the presented model, it was revealed that HBV DNA ≥20,000 IU/mL decreased the probability of HBeAg loss (OR = 0.19 (0.05–0.67)). Similarly, modelling for SRR showed that the passage of a month increased the odds of SRR achievement by approximately 3% (OR = 1.03 (1.01–1.06)). According to the definition of SRR (HBV DNA level <20 IU/mL, loss of HBeAg, and normalization of ALT for HBeAg (+) patients and HBV DNA <20 IU/mL and normalization of ALT for HBeAg(-) patients), quite understandably, the analyses of all patients showed that being HBeAg(+) may decrease the probability of the loss of SRR. However, in the HBeAg(+) subgroup, only time of therapy had a significant impact on the achievement of SRR. Analyses of the HBeAg(-) subgroup showed no covariates that affected the achievement of SRR. In order to better compare the SSR difference between HBeAg positive and negative patients, we excluded the HBeAg as a covariate from the GEE analysis. Resulting data showed that SRR in the whole group of patients was predicted by HBV DNA >20,000 IU/mL (*P* = 0.014, OR = 0.25 (0.08–0.76)), age at infection ≥10 years (*P* = 0.001, OR = 15.58 (3.62–67.00)) and time of therapy (*P* = 0.008, OR = 1.03 (1.0–1.05)). As we have shown previously, these variables were significant also for predicting the loss of HBV DNA for the whole group of patients. In the next step for this model, we analyzed prediction of SRR for HBeAg(-) and HBeAg(+) subgroups. We showed that just like in the model containing HBeAg as a covariate, only the time of therapy was significantly associated with SRR in the HBeAg(+) subgroup.

### Predictive on-treatment virological factors for treatment effectiveness

**Fig 3:**
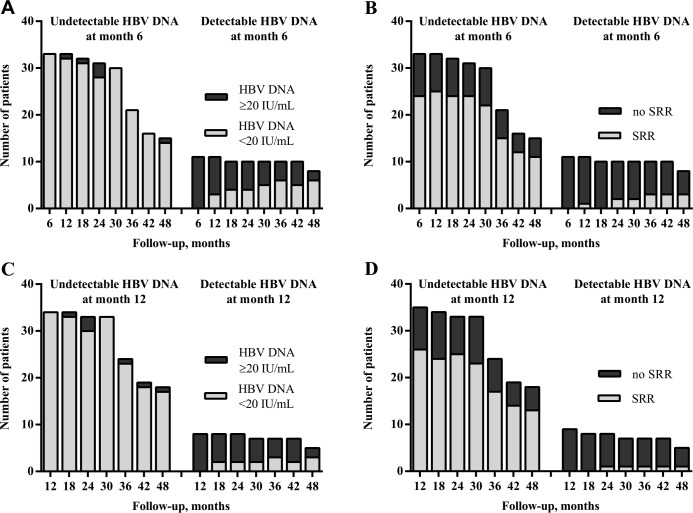
Distribution of CHB ETV-treated patients with undetectable HBV DNA and SRR stratified according to undetectable or detectable HBV DNA in months 6 and 12. HBV DNA suppression at month 6 determined the achievement of undetectable HBV DNA (A) and SRR (B) in the ensuing months of ETV therapy. Additionally, HBV DNA suppression at month 12 determined the occurrence of undetectable HBV DNA (C) and SRR achievement (D) in the ensuing months of ETV therapy.

An attempt to identify the most predictive factors of treatment effectiveness was made. Regardless of HBeAg status, it seemed that undetectable HBV DNA in month 6 or 12 predicted the achievement of undetectable HBV DNA (Fig 3A and 3C) and SRR (Fig 3B and 3D) in consecutive months of therapy. Patients with undetectable HBV at month 6 or 12 were more likely to reach undetectable HBV and SRR in later months of therapy than patients with HBV DNA ≥20 IU/mL at those time points. Several patients with HBV DNA ≥20 IU/mL in month 6 or 12 of therapy achieved virological suppression. However, a portion of them still had HBV DNA ≥20 IU/mL in months 36 and 48.

In the next step, we analysed the significance of the association of HBV DNA levels in months 6 and 12 with virological suppression and SRR in years 2, 3 and 4 (Table 5). Of the 44 patients, 75.0% (n = 33) achieved HBV DNA levels <20 IU/mL in 6 months of therapy. This was associated with virological suppression and SRR in years 1, 2 and 3, but not year 4, probably due to the insufficient size of the patient group undergoing treatment in year 4. All patients with HBV DNA <20 IU/mL at 6 months who had been treated for 36 months achieved undetectable HBV DNA at month 36. Patients with undetectable HBV DNA in their 6^th^ month of therapy had a 14 times higher odds (OR = 13.7, *P* = 0.002) of SRR achievement in the 24^th^ month of therapy than patients with HBV DNA ≥20 IU/mL in the 6^th^ month of therapy. Undetectable HBV DNA at month 12 was also associated with undetectable HBV DNA and achievement of SRR up to year 3. Patients with undetectable HBV DNA in their 12^th^ month of therapy had much higher odds of undetectable HBV DNA in their 36^th^ month of therapy than patients with detectable HBV DNA in the 12^th^ month of therapy (OR = 17.25, *P* = 0.007). Finally, undetectable HBV DNA in month 12 seemed to be a good predictor of achieving SRR in the 36^th^ month of therapy (OR = 14.57, *P* = 0.012).

**Table 5:** Significance of virological parameters in predicting long-term virological suppression and SRR among children and adolescent with CHB.

		Undetectable HBV DNA			SRR		
Parameter		n/total n (%)	*P* value	OR (CI)	n/total n (%)	*P* value	OR (CI)
Month 6 HBV DNA <20 IU/ml							
Month 12	32/33 (96.9)		<0.001	85.30 (7.83–93.26)	25/33 (75.8)	<0.001	31.25 (3.45–283.28)
Month 24	28/31 (90.3)		0.003	14.00 (2.46–79.55)	24/31(77.4)	0.002	13.71 (2.35–79.98)
Month 36	21/21 (100.0)		0.007	NA	15/21 (71.4)	0.048	5.83 (1.12–30.40)
Month 48	14/15 (93.3)		0.269		11/15 (73.3)	0.179	
Month 12 HBV DNA <20 IU/ml							
Month 24	30/33 (90.9)		<0.001	30.00 (4.09–119.99)	25/33 (75.7)	0.002	21.88 (2.33–105.78)
Month 36	23/24 (95.8)		0.007	17.25 (1.42–210.13)	17/24 (70.8)	0.012	14.57 (1.47–144.28)
Month 48	17/18 (94.4)		0.107		13/18 (72.2)	0.056	

Undetectable HBV DNA, HBV DNA <20 IU/ml; NA, not applicable

### Virological breakthrough and safety

Overall, in your study there were 5 patients who remained viremic at the end of observation. From this group, 4 patients (9.1%) developed virological breakthrough between month 30 and 48, 1 of whom had a coexisting biochemical breakthrough (defined as ALT > 2 times above the upper limit of normal (ULN) (40 IU/L) in patients who had normalized ALT and as ALT > 2 times the nadir in those who had never had normal ALT). Resistance profiling was determined among patients with detectable viremia during ETV therapy. ETV-resistant mutations observed as substitutions at positions T184, S202, M204 and L180 were present in 5 (11.4%) patients. Of this group, 3 (6.8%) patients developed virological breakthrough after 30 months of ETV therapy. The remaining 2 patients with ETV-resistant mutations did not develop virological breakthrough and were thus continued on therapy. In 1 of the 4 patients who developed virological breakthrough, resistant variants were not found. In the 4 patients with virological breakthrough, there were 3 patients previously exposed to LAM. One patient with virological breakthrough was naïve.

The patients in whom viremia (qualified as a virological breakthrough) was detected during the observation period were switched to therapy with tenofovir. If the viral load increased during the therapy—which was not qualified as a virological breakthrough—the patient continued therapy for 3–6 months, until the next inspection. In case of the one patient, the viral load at the end of the observation period (18 months) is still detectable, however, during to the constant drug administration, viremia was decreased to a level of 800 IU/mL whereby the patient continued therapy for 3–6 months, until the next inspection.

The treatment was safe and well tolerated. During antiviral treatment, there were no serious adverse events associated with the administered drug (Table 6). The most common treatment-related adverse events were associated with disorders of the digestive system. Six patients developed abdominal pain, two patients experienced vomiting and/or nausea and lack of appetite. Two patients developed a mild headache during the first weeks of treatment, which resolved without sequelae. No patients had decompensated liver function during the treatment requiring liver transplantation. In one patient, a mild ALT flare was observed. No significant difference in serum creatinine levels was observed between baseline and follow-up.

**Table 6:** Safety through 48 months of therapy among all ETV-treated patients.

		All patients (n, %)
N		44
SAEs		0 (0.0)
Any AE		13 (29.6)
	Abdominal pain	6 (13.6)
	Headache	2 (4.5)
	Pyrexia	1 (2.3)
	Vomiting/ nausea	2 (4.5)
	Rash	2 (4.5)
	Lack of appetite	2 (4.5)
	Facial palsy	1 (2.3)
	Numbness of the hands	1 (2.3)
	Menstrual disorder	1 (2.3)
	Dizziness	1 (2.3)
Laboratory abnormalities		
	ALT flares	1 (2.3)
	Leukopenia	2 (4.5)

SAEs—serious adverse events; AE—adverse event; ALT flares—defined as >2x ULN or >2x nadir

In our study we analyzed patient’s height, weight and BMI before the beginning of ETV therapy and at the end of the observation. Height, weight, and BMI were assessed using centile charts according to patient’s sex and age. By comparing the centile intervals distribution of height, weight and BMI from baseline to end of ETV treatment, we observed no impact of ETV therapy on expected growth (Table 7).

**Table 7:** The impact of ETV therapy on growth in CHB adolescents.

Growth			Centile intervals			
	<10	10–25	25–50	50–75	75–90	>90
Height						
before the start of the treatment	4 (9.1)	6 (13.6)	12 (27.3)	11 (25.0)	7 (15.9)	4 (9.1)
at the end of the observation	3 (6.8)	4 (9.1)	10 (22.7)	13 (29.6)	9 (20.4)	5 (11.4)
Weight						
before the start of the treatment	5 (11.4)	3 (6.8)	11 (25.0)	5 (11.4)	12 (27.3)	8 (18.2)
at the end of the observation	2 (4.5)	5 (11.4)	10 (22.7)	6 (13.6)	10 (22.7)	11 (25.0)
BMI						
before the start of the treatment	4 (9.1)	2 (4.5)	9 (20.5)	19 (43.2)	8 (18.2)	2 (4.5)
at the end of the observation	3 (6.8)	3 (6.8)	15 (34.1)	11 (25.0)	9 (20.5)	3 (6.8)

## Discussion

Despite the enhanced treatments for HBV infection in children and adolescents, improved by the optimization of existing treatment programs and the introduction of new drugs for therapy, currently we can speak at most of the half success of currently available therapies aiming at stop of progression of the underlying liver injury [8, 10].

To the best of our knowledge, this is a unique longitudinal study that specifically examines the long-term efficacy of ETV treatment in adolescents in real life. Until now, no data regarding the long-term efficacy of ETV monotherapy for the treatment of CHB in children and adolescents have been presented. In fact, there is limited information regarding the results of ETV monotherapy in children and adolescents infected with CHB, restricted by the small size of the test groups and the limited duration of ETV therapy. A study conducted by Saadah et al. reported retrospectively the efficacy of ETV treatment in 8 HBeAg(+) CHB children, including both those who were previously treated with lamivudine and those who were treatment-naive. The mean duration of ETV treatment was 24 months. In those patients, HBV DNA levels were undetectable in 37.5%, with ALT normalization in 87.5% and HBeAg seroconversion in 37.5% [11]. In our previous study conducted with 30 children, we showed that 24 weeks of treatment with ETV results in the suppression of HBV DNA in 40.0% of children with CHB who had been previously treated ineffectively [12]. Chu et al. revealed that 48-week ETV monotherapy in 8 children with lamivudine-resistant CHB exhibited a significantly more effective virologic response than monotherapy with adefovir [13]. In Chang et al.’s study, which enrolled 9 treatment-naive HBeAg-positive patients, undetectable HBV DNA levels and cumulative incidence rates of HBeAg seroconversion at 1 year of ETV treatment were observed in 55.6% and 44% patients, respectively [14]. In the current year, the first results of an ongoing, randomized phase III study conducted on a large sample of 120 CHB HBeAg-positive nucleos(t)ide-naive children aged 2 to <18 years were provided [15]. That study assessed the safety and efficacy of 48-week monotherapy with entecavir. Virological suppression (HBV DNA <50 IU/mL) and HBeAg seroconversion at week 48 of therapy were achieved in 49.2% and 24.2% of patients, respectively. Similarly, in our study, HBV DNA <20 IU/mL and HBeAg seroconversion at week 48 of therapy were achieved by 65.2% and 30.4% of the HBeAg-positive patients, respectively. Overall, the data from our study demonstrated high cumulative rates of HBV DNA <20 IU/mL (89.7%) and HBeAg seroconversion (55.4%) and a high percentage of ALT normalization (95.45%) in entecavir-treated adolescents in year 4.

Our data concerning the virological, biochemical and serological suppression during long-term ETV treatment in adolescents are in accordance with the numerous studies in adults showing high rates of outcomes. The results of a study among 533 CHB patients treated with ETV demonstrated that the rate of undetectable HBV DNA increased steadily from year 1 to 3, reaching 91.2% [16]. In a study by Chang *et al*. assessing ETV therapy in adults, at year 5, 94% of patients had HBV DNA levels <300 copies/mL, and in 80%, ALT activity was normal [17]. Luo et al. analysed the efficacy and safety of ETV treatment for up to 5 years in real life in 230 nucleos(t)ide-naïve CHB patients and revealed an increase in rates of undetectable serum HBV DNA from 67.0% at month 3 to 100% after 5 years. The rate of patients achieving ALT activity normalization increased from 73.9% to 100% during the same time frame [18]. Yan et al. evaluated the antiviral response of 99 HBeAg-positive CHB patients after 96 weeks of ETV treatment. A virological response (VR), defined as HBV DNA <300 copies/mL, was achieved in 42%, 62%, and 68% of high viral load (HVL—HBV DNA >9 log 10 copies/mL) patients and in 67.34%, 85.71%, and 85.71% of non-high viral load patients at weeks 48, 72, and 96, respectively [19]. In an Ono *et al*. study among 474 patients with CHB, by the fourth year of treatment, increases in the rates of undetectable HBV DNA and ALT activity normalization up to 96% and 93% of patients, respectively, were observed [20].

HBV DNA suppression is a primary goal of therapy for CHB. The relationship between baseline and on-treatment factors and ETV treatment outcomes has not been widely studied in paediatric patients. Regarding the most predictive factor, undetectable HBV DNA in the 12^th^ month of treatment was selected as the best predictor of assessed virological suppression; however, the results of our study demonstrated that undetectable HBV DNA in the 6^th^ month of treatment can also be considered an early predictive factor of virological outcomes up to year 3 of ETV therapy. In our study, undetectable levels of HBV DNA in the 36^th^ month of therapy were identified in 21/21 (100%) and 23/24 (95.8%) patients who had undetectable HBV DNA at months 6 and 12 of therapy, respectively. These data are also consistent with previous findings in adults. In a multivariate analysis, HBV DNA level at month 12 was identified as an independent predictor of undetectable HBV DNA in the third year of therapy [20]. However, our observations suggest that some patients require more time to reach HBV DNA clearance and that detectable HBV DNA at the 6^th^ or 12^th^ month does not necessarily indicate unsuccessful treatment, although it can. Zoutendijk et al. revealed that NA-naïve patients with a partial response to ETV may benefit from prolonged therapy without the development of resistance [21]. Moreover, our study revealed that patients with undetectable HBV in the 6^th^ or 12^th^ month of therapy are also more likely to achieve SRR in years 2 and 3 of therapy than patients with HBV DNA ≥20 IU/mL. These results are consistent with those presented by Wong *at al*. for NA-naïve adults, who found that an undetectable HBV DNA level at month 12 of ETV therapy was an independent predictor of virological and serological suppression during 3 years of treatment [22].

Information regarding the long-term HBV DNA kinetics during treatment with NAs in adolescent is limited. The analysis performed in this study revealed that the probability of the loss of HBV DNA is associated with the length of the treatment period and that the passage of each month during therapy increases the odds of the loss of HBV DNA by approximately 5%. The passage of one year of therapy more than doubles the odds of the loss of HBV DNA. Patients with HBeAg(+) CHB at baseline have, on average, approximately 80% lower odds of the loss of HBV DNA than patients who are HBeAg(-) at baseline, at any time point.

In our retrospective study, the patients were referred for ETV treatment only if there were no mutations predisposing them for acquiring entecavir resistance before treatment. GEE analyses include a variable indicating the presence of previous LAM treatment, which proved to be irrelevant. We can assume that this is related to the fact that patients with mutations resulting from the administration of LAM did not take part in the study. However, this is currently the therapeutic standard, developed on the basis of previous clinical trials and observational studies conducted in groups of patients previously treated with LAM. Univariate analysis showed no association of previous NA-therapy with baseline factors like: gender, age, ALT activity and HBV DNA viral load with cut off 2,000 IU/mL. Only for HBV DNA ≥20,000 IU/mL we noted significant association (*P* = 0.031). We can suppose that the lack of a link between previous LAM treatment and the therapy results determined by GEE analysis, is due to the more significant impact of HBV DNA ≥20,000 IU/mL variable on the final therapeutic effect. Moreover, in our study the proportion of mutations resulting from the administration of ETV was relatively low, was not always associated with previous LAM treatment, did not always have an impact on the virological supression, and therefore the previous NA-therapy variable was not relevant in predicting the effectiveness of treatment. In our study, 4 of the 5 patients with entecavir resistance had prior LAM exposure, but only 2 of them developed virological breakthrough.

Interestingly, the GEE model also shows a potential association between older infection age (≥10 years) and the loss of HBV DNA; higher infection ages increased the probability of the loss of HBV DNA. These results seemed to confirm the opinion that it is not appropriate to treat younger children in the immune-tolerant phase. Age at the start of treatment appeared to be non-significant in the model; therefore, no differences in the evolution of HBV DNA loss during treatment in adolescents and in adults has been detected, and it seems that the treatment of adolescents is as effective as it is in adults. Despite the frequent inability to determine the age of infection in clinical practice, the significance of the higher infection age in ETV therapy needs to be assessed in large studies.

The results of the available studies and clinical trials suggest that entecavir was well tolerated [11, 14, 15]. However, the safety of entecavir in children should be demonstrated through a long-term study that enrols a relatively large number of patients. In our study, entecavir was safe, with no serious adverse events reported up to year 4, which is consistent with previous results. Resulting data showed that growth was not compromised by exposure to entecavir therapy. In our study, there was one asymptotic mild ALT flare that occurred during treatment, which was associated with virologic breakthrough and genotypic resistance. Overall, 5 patients developed entecavir resistance, 3 of whom developed virological breakthrough. Four of the 5 patients with entecavir resistance had prior LAM exposure. The remaining patient was naive. This observation suggests that resistance may be an important factor impeding virological suppression in the LAM-exposed subgroup.

There are several limitations to this study, including the relatively small sample size of the treatment subgroups. Another limitation is the heterogeneous population of adolescents, including patients with different previous treatment histories, HBeAg status and baseline ALT activity. Most of the currently available studies on NA therapy efficacy in children and adolescents have this limitation. However, by stratifying according to HBeAg status and using GEE analyses for longitudinal studies, we could evaluate the importance of individual differences at baseline on the long-term outcomes. Multicentre clinical trials with ETV therapy in children and adolescents are ongoing [23, 24], and other long-term follow-up studies are needed.

## Conclusions

These results support ETV as an effective and safe treatment option for children and adolescents with CHB. Extended therapy with ETV leads to high rates of HBV DNA suppression, HBeAg seroconversion and ALT activity normalization. Patients who maintained virological suppression could be predicted by having undetectable levels of HBV DNA in the 6th and/or 12th month of ETV therapy. This study revealed the role of age at infection in predicting virological suppression after ETV therapy in children and adolescents chronically infected with HBV. Thus, knowledge of the age at infection may be useful in clinical practice in enhancing the prediction of long-term outcomes in ETV-treated children and adolescents.
